# A study on the effectiveness of pharmacopuncture for chronic neck pain: A protocol for a pragmatic randomized controlled trial

**DOI:** 10.1097/MD.0000000000021406

**Published:** 2020-07-31

**Authors:** Kyoung Sun Park, Yoon Jae Lee, Jinho Lee, In-Hyuk Ha

**Affiliations:** a Jaseng Hospital of Korean Medicine; b Jaseng Spine and Joint Research Institute, Jaseng Medical Foundation, Gangnam-gu, Seoul, Republic of Korea.

**Keywords:** chronic neck pain, pharmacopuncture, physical therapy, pragmatic randomized controlled trial, protocol

## Abstract

**Background:**

Neck pain is a common musculoskeletal disorder that impacts individuals’ daily life, and might sometimes lead to disability and increased medical costs. Pharmacopuncture combines acupuncture with herbal medicine, in which herbal extracts are administered on the acupoints. We designed a pragmatic randomized controlled trial (RCT) to compare the effectiveness of pharmacopuncture and physical therapy as a treatment for chronic neck pain.

**Methods:**

This study is a 2-armed, parallel, multi-center RCT. The participants will be randomly assigned to pharmacopuncture or physical therapy group to receive their respective 8-session treatments for 4 weeks. The primary outcome is the visual analogue scale of neck pain. Secondary outcomes is the Northwick Park questionnaire, visual analogue scale of radiating arm pain, numeric rating scale of the neck and arm bothersomeness, neck disability index, patient global impression of change, short form-12 health survey version 2, and 5-level EuroQol-5 dimension.

**Discussion:**

This is a protocol for a pragmatic RCT that would attempt to present evidence conducive to clinical decision or policy-making by investigating the effectiveness of pharmacopuncture therapy, a widely used approach in Korean medicine clinical practice, in comparison to the standard therapy.

## Abstract 2

Supplemental Digital Content is available in the text

## 1 Introduction

Neck pain is a common musculoskeletal disorder that impacts individuals’ daily lives. It might sometimes lead to disabilities and an increase in medical costs. The 2010 Global Burden of Disease ranked neck pain as the fourth major factor of disability, following back pain, major depressive disorder, and other musculoskeletal disorders.^[1]^ According to a 2006 systematic review of neck pain, its prevalence in one's lifetime in adults (18–84 years) worldwide was about 50%. There are, however, regional differences,^[2]^ and women were reported to be more frequently affected by neck pain than men.^[2–4]^ The primary treatments for neck pain are oral or injected medication. While non-steroidal anti-inflammatory drugs are the most effective agents,^[5]^ they can cause adverse reactions such as gastritis, gastric ulcers, gastrointestinal bleeding, and myocardial infarctions.^[6]^

Pharmacopuncture combines acupuncture with herbal medicine, in which herbal extracts are administered on the acupoints. It can maximize and extend the effects of acupuncture by optimizing the acupoint access through an integration of physical stimulation and chemical effects.^[7,8]^ A 2016 study examined the frequency and details of different traditional treatment approaches performed in 12 Korean medicine institutions. The study found that 32,947 (98.6%) among 33,145 inpatients and 289,860 (77.6%) among 373,755 outpatients received pharmacopuncture therapy.^[9]^ A 2015 questionnaire survey of Korean medicine doctors, affiliated with Korean medicine institutions specializing in musculoskeletal disorders, reported that 95.9% of the respondents (118 out of 123) utilize pharmacopuncture for neck pain.^[10]^ According to a recent study on the status of pharmacopuncture procedures for neck pain in Korea, the most commonly performed procedure was bee venom pharmacopuncture, followed by Flos Carthami pharmacopuncture, Scolopendra pharmacopuncture, Ouhyul pharmacopuncture, Hwangryun pharmacopuncture, Corpus pharmacopuncture, Soyeom pharmacopuncture, Hwangryun-haedok-tang pharmacopuncture, and Shinbaro phamacopuncture.^[11]^

A systematic review and meta-analysis on the effects of pharmacopuncture on neck pain reported that pharmacopuncture monotherapy and pharmacopuncture-acupuncture combination therapy are significantly more effective in reducing the severity of neck pain and increasing the quality of life than acupuncture monotherapy or physical therapy (PT).^[12]^ However, existing randomized controlled trials (RCTs) on pharmacopuncture are highly heterogeneous and with a low level of evidence. It is, therefore, impossible to draw concrete conclusions on the effectiveness of pharmacopuncture therapy. A well-designed RCT that reflects the clinical context in Korea is thus needed. With this in mind, we planned a pragmatic RCT that aims to compare the effectiveness of pharmacopuncture and PT therapies on chronic neck pain.

## 2 Methods

### 2.1 Study design and setting

This study is a 2-armed, parallel, multi-center RCT that will be performed on 100 patients in the following institutions: Jaseng Hospital of Korean Medicine, Daejeon Jaseng Hospital of Korean Medicine, Bucheon Jaseng Hospital of Korean Medicine, and Haeundae Jaseng Hospital of Korean Medicine. Participants will be enrolled via competitive recruitment. The study protocol was approved by the institutional review board (IRB) at each institution before starting the participant recruitment process (JASENG 2019-06-009, JASENG 2019-06-010, JASENG 2019-06-011, and JASENG 2019-06-008). Further, the protocol has been registered with Clinicaltrials.gov (NCT04035018) and Clinical Research Information Service (KCT0004243) for continuous updates on the study progress. Information about the healthcare institutions and investigators can be found on the trial registration website.

### 2.2 Participant timeline

On the first visit, the participants will sign an informed consent form (ICF) after receiving an explanation about the study. The examiner will screen the participants for eligibility per the inclusion/exclusion criteria. The participants will be randomized to the pharmacopuncture or the PT group during the second visit and undergo their respective treatments thereafter. The recruited participants will visit the facility 13 times, 8 of which will be treatment sessions (twice per week for 4 weeks). In-person or over-the-phone follow-up surveys will be conducted on weeks 5, 8, and 12 after treatment initiation. The schedule of enrollment, interventions, and assessments for the participants is shown in Table 1.

**Table 1:**
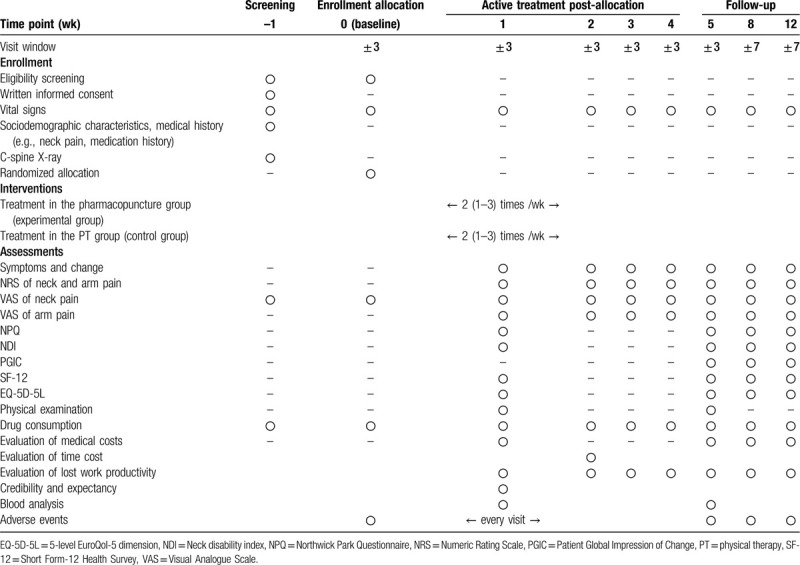
The schedule of participants.

### 2.3 Inclusion/exclusion criteria

**Inclusion criteria**

1) Neck pain for more than 6 months.
2) Visual analogue scale (VAS) of neck pain more than 5.
3) Age between 17 and 70 years.
4) Signed informed consent.

**Exclusion criteria**

1) Patients having any of the following:
2) migration of cancer reaching the spine or a fracture of the spine.
3) Progressive or severe neurologic deficits.
4) Cancer, fibromyalgia, rheumatoid arthritis, or gout.
5) Hemorrhagic disease, stroke, myocardial infarction, kidney disease, dementia, severe diabetes or diabetic neuropathy, or epilepsy.
6) Taking steroids, immunosuppressants, anticoagulants, or psychotropic medication.
7) Taking non-steroidal anti-inflammatory drugs or receiving pharmacopuncture within the past week.
8) Pregnant or lactating women.
9) Undergone cervical surgery within the past 3 months.
10) Participated in another clinical trial within the past month, or plan to participate in another trial during the follow-up period of this trial.
11) Cannot write an informed consent.
12) Has other difficulties in participating according to the investigator's decision.

### 2.4 Interventions

#### 2.4.1 Experimental group: pharmacopuncture

Pharmacopuncture therapy will be performed twice weekly for 4 weeks. One additional or fewer session per week would be allowed, depending on the patient's status. In other words, a minimum of 1 session and a maximum of 3 sessions can be performed each week. Three sessions per week might be needed in the earlier stages, during which the patient's symptoms might be severe. One session per week might suffice once symptoms have improved. The total number of sessions will not be set in advance. Rather, the physician in charge can make the clinical decisions depending on the patient's status. All procedure-related matters will be recorded in the medical chart for accurate assessment, following the methodology of a study that has analyzed the current use of pharmacopuncture therapy based on the electronic medical records in 12 Korean medicine hospitals.^[9]^ All pharmacopuncture therapies used will be retrospectively reviewed and recorded in the case report form (CRF) for analysis.

#### 2.4.2 Control group: PT

A review article that used data from the Korean health insurance review and assessment service found that various types of PT (e.g., superficial heat therapy, deep heat therapy, traction, electrotherapy) are combined in clinical practice, depending on the patient's symptoms.^[13]^ Based on these data, we will allow the physician to make the clinical decisions about the type, site, and duration of PT, depending on the patient's symptoms, magnetic resonance imaging data, and degree of improvement. PT will be performed twice weekly for 4 weeks. One additional or fewer session per week would be allowed, depending on the patient's status. In other words, a minimum of 1 session and a maximum of 3 sessions can be performed each week. Three sessions per week might be needed in the earlier stages during which the patient's symptoms might be severe. One session per week might suffice once symptoms have improved. The number of sessions will be determined by the prescribing physician, depending on the patient's status. All matters pertinent to the type, duration, and site of PT will be recorded in the patient's chart for an accurate assessment. All types of treatment used will be retrospectively reviewed and recorded in the CRF for analysis.

### 2.5 Criteria for discontinuation and withdrawal

The trial can be discontinued for a participant under the following conditions:

1) discovery of an illness that had not been detected at the baseline assessment and might impact the evaluation of the study results.
2) Request to discontinue the study by the participant or by the participant's legal representative, or withdrawal of the consent by the participant during the study period.
3) Confirmation of pregnancy during the study.
4) Presence of problems related to undergoing medical or Korean medical procedures for neck pain.
5) Other conditions in which the continuation of participation in the study is deemed inappropriate by the investigator in charge.

### 2.6 Concomitant treatment

Patients can seek drug therapy and care at another healthcare institution in cases of severe pain during the treatment or the follow-up period. The details and frequency of such treatments will be recorded thoroughly in the CRF and used for the analysis.

### 2.7 Outcomes

#### 2.7.1 Primary outcome: VAS of neck pain

The VAS is one of the means for estimating the patient's pain. It is an instrument consisting of a straight 100-mm line with the left-most point indicating no pain and the right-most point indicating the most excruciating pain one can imagine. Each patient would mark the position that best represents the level of her or his pain. At each assessment, the patients would mark a point on the line that represents their neck pain during the preceding week.

#### 2.7.2 Secondary outcome

##### 2.7.2.1 Northwick Park questionnaire (NPQ)^[14]^

The NPQ is a self-reported questionnaire, consisting of 9 items about daily life activities that are influenced by neck pain (intensity, sleep, numbness, duration, carrying things, reading/watching TV, working, social activities, and driving). Each item consists of 1 question and 5 responses that indicate growing levels of difficulty and pain rated from 0 to 4, with 4 indicating the most significant functional impairment. The total score is a sum of the scores for all 9 items. In the present study, we will use the Korean version of the NPQ, which was translated and validated by Lee et al in 2010.^[15]^

##### 2.7.2.2 VAS of pain radiating to the arm

The VAS, as described above, will be used to assess the patients’ pain radiating to the arm. The patients would mark a point on the line that represents their radiating arm pain during the preceding week.

##### 2.7.2.3 Numeric rating scale (NRS) of neck and arm bothersomeness

The intensity of discomfort in the neck and upper arm area during the past week will be assessed using the NRS. Patients will choose a number between 0 to 10 that best represents their level of discomfort, with 0 being no pain and 10 being the most excruciating pain one can imagine.

##### 2.7.2.4 Neck disability index (NDI)^[16]^

The NDI was developed to examine the level of disability caused by neck pain in daily life. Ten items are scored, with each item being rated from 0 to 5. The NDI is calculated by dividing the total score by the number of scored items.

##### 2.7.2.5 Patient global impression of change^[17]^

The Patient global impression of change is a subjective evaluation of the level of improvement by the patients:

1. very much improved
2. much improved
3. minimally improved
4. no change
5. 5 minimally worse
6. much worse
7. very much worse

##### 2.7.2.6 Short form-12 health survey (SF-12) version 2

The SF-12 is a questionnaire for assessing health-related quality of life. It consists of 12 items in 8 domains: physical functioning, role-physical, bodily pain, general health, vitality, social functioning, role-emotional, and mental health. It takes less than 5 minutes to complete the survey, and a higher score indicates a better health-related quality of life. In the present study, we will use a validated Korean version of the SF-12.^[18]^

##### 2.7.2.7 5-level EuroQol-5 dimensions (EQ-5D-5L)

The EQ-5D-5L is an indirect measure of the quality weights of particular health conditions by appraising health status from multidimensional aspects and using the pre-designated preference weights for each functional level. It is the most widely used indirect measure instrument. The EQ-5D-5L is comprised of 5 dimensions (mobility, self-care, usual activities, pain, anxiety/depression), and the level of each dimension is surveyed. Weighted values are designated according to each dimension's level, and the preference score is calculated based on these weights and specific constants.^[19]^

##### 2.7.2.8 Costs data

To measure costs, we will use a survey, comprising of items that will estimate direct medical costs (healthcare service), direct non-medical costs (purchasing foods, medical devices, and transportation fee), and indirect costs (time cost and loss of productivity). Loss of productivity are defined as the financial loss caused by the inability to work due to the disease itself or premature death from the disease. Loss of productivity will be measured using the work productivity and activity impairment questionnaire,^[20]^ and the value will be converted to a cost to be used in the cost-effectiveness analysis.

##### 2.7.2.9 Drug consumption

The types and doses of drugs (rescue drugs) that the participants took for neck pain during the study period will be identified via a survey during their respective visits. Other types of therapies, such as other PT and injections, will be recorded as if they were sessions received.

### 2.8 Adverse events

For safety assessment, white blood cell, neutrophil, lymphocyte, monocyte, eosinophil, basophil, red blood cell, hemoglobin, hematocrit, mean corpuscular volume, mean corpuscular hemoglobin, mean corpuscular hemoglobin concentration, platelet, erythrocyte sedimentation rate, total protein, albumin, total bilirubin, alkaline phosphatase, aspartate aminotransferase, alanine aminotransferase, gamma-glutamyl transpeptidase, and C-reactive protein will be performed before the start and after the end of treatment sessions in both study groups to compare the incidence of adverse events between groups.

An adverse event refers to undesirable and unintended signs, symptoms, or diseases that would occur after a procedure. It includes responses that are not directly related to the procedure. In the present study, we will collect data on adverse events based on the patients’ reports of symptoms and our observations. We will then analyze the incidence of adverse events that are suspected to be related to the treatment. This would include abnormal laboratory results and serious adverse events. The causality between the treatment and the adverse events will be assessed based on a 6-point scale, following the world health organization-Uppsala monitoring center causality assessment system (1 = definitely related, 2 = probably related, 3 = possibly related, 4 = probably not related, 5 = definitely not related, and 6 = unknown). The severity of all adverse events will be classified into 3 levels following the Spilker classifications:

(1) Mild, does not need treatment or does not significantly impair daily activities (functions)
(2) Moderate, significantly impairs daily activities (functions) and may require treatment but resolves afterward
(3) Severe, a serious adverse event that requires intensive treatment and leaves sequelae.

### 2.9 Sample size calculation

The null hypothesis of this study is that there will be no difference in the outcomes between the experimental and control groups when treating chronic neck pain. The hypothesis will be tested using analysis of covariance (ANCOVA) as the primary analytical test. The significance level will be set to α = 0.05 (2-tailed), with type 2 error (β) of 0.2 and a power of 80%. The sample required size was calculated based on a meta-analysis that had analyzed the effectiveness of pharmacopuncture compared to acupuncture or electropuncture therapy in patients with cervical spondylosis.^[12]^ Although the control group of this study differs from ours, we determined that we can utilize that study for sample size calculation based on their findings that the effectiveness of acupuncture in treating neck pain is equivalent^[21]^ or superior^[21,22]^ to that of PT. The mean difference in VAS between the pharmacopuncture and control groups based on this meta-analysis was 1.79 ± 2.74.^[12]^ When effect size is calculated based on this, the f-value is 0.33. Using the G∗Power software, version 3.1.9.4 (Franz Faul, Christian-Albrechts-Universität Kiel, Kiel, Germany), a total of 77 participants will be required. Assuming a withdrawal rate of 20%, we plan to recruit 100 participants.

### 2.10 Recruitment

Study participants will be recruited through online media releases, subway advertisements, and promotional posters posted inside and outside the study sites.

### 2.11 Randomization and allocation concealment

A statistical expert will randomize an equal number of patients (n = 50, each) into 2 groups, using a random table generated by the nQuery Advisor (Statistical Solutions Ltd., Cork, Ireland) 7.0 (or SAS 9.0 (SAS institute, Cary, USA) or SPSS 21.0 (SPSS Inc., Chicago, USA)) software. The randomization results will be delivered to each institution in sealed opaque envelopes to be stored in a double-locked cabinet. The investigator will provide an adequate explanation of the trial, and each patient that meet the inclusion and exclusion criteria and voluntarily sign the consent form will open a randomization envelope to be randomly assigned a subject number. The investigator will record the assigned random number for each patient on the patient's electronic chart.

### 2.12 Blinding

As it is impossible to blind the physicians delivering the intervention and participants receiving the intervention due to the nature of the interventions, we will only blind the assessors. The resident Essessors, blinded to the participants’ allocation and not involved in the intervention, will perform the evaluations of each outcomes in a separate place, prior to the interventions.

### 2.13 Data collection and management

We will keep in touch with the participants from recruitment to 3 months after treatment to promote participant retention and complete follow-up. The patients will be able to contact researchers at any time to get information they need.

This study will utilize electronic CRF (e-CRF) using the internet-based Clinical Research and Trial management system, managed by the Korea Centers for Disease Control and Prevention. The standard operating procedure (SOP) will be distributed for reference to the study-related procedures, including writing the CRF, entering data, and educating the evaluator and investigators at each site. A data query will be issued for range checking of the data values. Data entered in the e-CRF will be cleaned, locked, and concealed from all investigators, except for the investigator in charge of data management.

### 2.14 Statistical methods

Sociodemographic characteristics and treatment expectancy will be evaluated for each group. Continuous variables will be expressed as mean (standard deviation) or median (quartiles). Differences between the 2 groups will be analyzed using the Student *t* test. Categorical variables will be expressed as frequency (%) and will be analyzed using the Chi-squared test or Fisher exact test.

The endpoint of this clinical trial is the differences in changes in the continuous outcomes (NRS, VAS, NDI, NPQ, EQ-5D-5L, SF-12) at each time point compared to the baseline between the 2 groups. As the primary analysis, ANCOVA will be performed with factors that significantly differ between the 2 groups at baseline as covariates and the groups as fixed factors. As an additional analysis, a linear mixed model will be used to analyze the differences in the changes between visits and the trend of such changes. The linear mixed model will be processed with the mixed model for repeated measures. In addition, the areas under the curve (AUC) will be calculated at each time point after randomization for each outcome during the treatment period and through the entire study period. The Student *t* test will be used to compare the difference in AUC between 2 groups. Additionally, percentages of patients whose NRS and VAS for neck pain decreased by more than 50% from the baseline will be compared for each time point. The time since randomization to the improvement of neck pain, as determined by a drop in the pain indices by more than 50%, will be estimated using the Kaplan–Meier survival analysis. The curves will be compared using the log-rank test. Furthermore, hazard ratios will be compared using the Cox proportional hazard model.

In this study, we will perform both intention-to-treat (ITT) and per-protocol analyses, with ITT analysis as the primary approach. For PP analysis, participants who underwent 6 or more treatment sessions during the 4-week treatment period will be analyzed separately. Missing data in the principal ANCOVA analysis and the AUC analysis will be processed by multiple imputation. Missing time point data in the survival analysis will be censored. All statistical analyses will be performed using the SAS version 9.4 statistical package (SAS Institute, Cary, USA), and differences will be considered statistically significant when *P* < .05.

### 2.15 Data monitoring

The data monitoring will be performed to examine the safety of participants and the clinical data's completeness. This monitoring will be done by comparing the CRF and the source documents and by reviewing participants’ safety data. Routine monitoring visit will be conducted 4 times, and closing-out visit will be done once at each site during the trial. Trial steering group will meet to review conduct throughout the trial period once every 2 months. The data monitoring committee was not organized as no severe AE regarding pharmacopuncture or PT previously reported are expected to occur. The interim analysis will not be done in this study.

### 2.16 Stopping rules

The trial might be discontinued at any point upon discovery of an unexpected, evident or unacceptable risk for participants, or upon the occurrence of serious adverse events, presumed to be related to pharmacopuncture or PT, in more than 25% of the participants.^[23]^ The PI would make the final decision if to discontinue the study.

### 2.17 Harms

To evaluate the safety, both the pharmacopuncture and PT groups will undergo blood test (white blood cell, neutrophil, lymphocyte, monocyte, eosinophil, basophil, red blood cell, hemoglobin, hematocrit, mean corpuscular volume, mean corpuscular hemoglobin, mean corpuscular hemoglobin concentration, platelet, erythrocyte sedimentation rate, total protein, albumin, total bilirubin, alkaline phosphatase, aspartate aminotransferase, alanine aminotransferase, gamma-glutamyl transpeptidase, and C-reactive protein) before treatments initiation and after the last treatment. The collected blood samples will be discarded immediately after analyzing the results. Matters pertinent to biological samples disposal will adhere to the SOP presented by the medical laboratory team. All adverse events reported during the study period will be collected, and the percentage of patients who develop an adverse event will be calculated for each group. The incidence of adverse event in the 2 groups would then be compared using the Chi-squared test or Fisher exact test.

### 2.18 Auditing

Audit will be conducted at each trial site once during the trial by investigators independent from this study.

### 2.19 Ethical approval

Before the start of the study, the study protocol, CRF, ICF, and patient recruitment announcement have been submitted to the IRB at each site and obtained approval for the study (JASENG June 06, 2019, JASENG June 10, 2019, JASENG June 11, 2019, and JASENG June 08, 2019). Any amendments to the protocol, CRF, ICF, or patient recruitment announcement will be made only upon IRB approval. All changes will be updated on the trial registries. All clinical investigators participating in this study will be educated about the Helsinki Declaration and the Korean Good Clinical Practice Guidelines, the study protocol, and the SOP to protect the patients participating in this study.

### 2.20 Informed consent

Before the start of the clinical trial, investigators will provide the patients with an adequate explanation about the contents of this clinical trial and information about the treatments. The investigators would obtain a signed informed consent and provide a copy of the consent form to each participant. If personal information would need to be collected, such as a copy of a bankbook to receive the transportation reimbursement, we will explain this need to the patients and obtain their consent for collecting personal information.

### 2.21 Confidentiality

All personal information of trial participants will be strictly managed under IRB supervision, and confidentiality of personal data will be strictly protected. All data collected from participants who consented to participate in this trial would be processed anonymously. If such data were to be disclosed to a third party for research purposes, arbitrary codes would be used with all personal identifying information removed.

### 2.22 Ancillary and post-trial care

An emergency contact number will be provided to all trial participants to contact in case of questions, when experiencing medical problems, or a study-related symptom during the study period, so they can receive the necessary assistance. Any participant directly harmed in relation to the trial would be legible to receive appropriate medical care as determined by the investigator. Compensations for any damages would be processed per the designated trial-related insurance agreement.

## 3 Discussion

Pragmatic clinical studies have been developed based on the perspective that the conventional explanatory trials do not produce appropriate clinical outcomes because they are optimized to determine efficacy. Highly trained investigators have conducted explanatory trials on relatively small sample size and strictly selected patient groups.^[24]^ Schwartz and Lellouch stated that explanatory trials are studies that test a physiological or clinical hypothesis. In contrast, pragmatic trials are studies that contribute to clinical decision or policy-making by providing evidence for selecting an intervention in a real-world practice.^[25]^ In other words, pragmatic trials have been designed to overcome the limitations of conventional clinical trials. Their significance is in determining treatment outcomes in real-world clinical settings. Notably, many Korean medicine trials are focused on verifying the effectiveness of clinically popular treatment or comparing their effectiveness with that of other treatments, as opposed to developing or aiming to obtain approval of novel drugs or medical technologies. Pragmatic clinical trial models are, therefore, more appropriate in studies related to Korean medicine.

A pragmatic-explanatory continuum indicator summary (PRECIS) is a tool with 10 domains that helps design clinical trials on a continuum from explanatory attitude (ideal situation) to a more pragmatic attitude (usual care).^[26]^ However, weaknesses have been highlighted: the absence of a rating scale, problems with some domains, need for better guidance, and lack of validation. The revised version of PRECIS, PRECIS-2, has 9 domains: eligibility criteria, recruitment, setting, organization, flexibility (delivery), flexibility (adherence), follow-up, primary outcome, and primary analysis, which scored from 1 (very explanatory) to 5 (very pragmatic).^[27]^

In this study, we will be recruiting a broad spectrum of patients with chronic neck pain, irrespective of imaging results, but only between the ages of 17 and 70 years. The eligibility criteria can, therefore, be given a score of 4. Recruitment is given a score of 4 as it will be utilizing means such as subway advertisements to approach a wider audience. The setting could be given a score of 3, as we will only be recruiting participants at 4 hospital-level healthcare institutions. The organization could be assigned a score of 5 for not relying on imaging equipment or assistance from other experts in the diagnosis and treatment of neck pain. Flexibility (delivery) could also be given a score of 5 because we will not be designating a specific treatment regimen for the physicians to follow. Rather, we will be providing data on the frequently used pharmacopuncture and PT regimens for an outpatient setting,^[9,13]^ and let the physicians autonomously choose, deliver, and chart the type and site of treatment within the confinements of their respective treatment group, which will be retrospectively analysed. Such clinical decisions will be made based on the patients’ symptoms and imaging results. Flexibility (adherence) could be given a score of 4. Although participants will not be dropped out based on a specific adherence criterion during the treatment period, we will recruit patients who can visit the facility for an adequate number of treatment sessions during the participant screening stage. Follow-up can be given a score of 2 because a variety of questionnaires will need to be completed during over-the-phone or in-person follow-up sessions at designated time points after the completion of treatment. This would require additional time and subject participation beyond the general treatment. The primary outcome domain could be given a score of 5, as the primary outcome of this study is VAS for neck pain. This primary outcome is directly related to the patient's symptoms and is an important outcome from the patient's perspective. The primary analysis could also be given a score of 5. We plan to perform ITT analysis using data from all participants, including those who showed noncompliance and those with values below the expected level. The PRECIS-2 wheel of this protocol is shown in Supplementary 1.

This protocol delineates a pragmatic RCT that aims to compare the effectiveness of pharmacopuncture, an intervention widely used in Korean medicine clinical practice, with that of the standard treatment. Our goal is to present evidence that would assist clinical decision and policy-making.

### 3.1 Protocol version

The study protocol version is 1.5 (March 16, 2020). Important amendments to the study protocol or other changes hereafter will be periodically updated on the trial registration sites.

### 3.2 Dissemination policy

The investigators will share the results of this study with the participants, healthcare professionals, and the public by publishing or through trial registries. Only investigators who were directly or indirectly involved in the study will be listed as authors on study-related publications.

## Author contributions

**Conceptualization:** Kyoung Sun Park, In-Hyuk Ha.

**Methodology:** Kyoung Sun Park, Yoon Jae Lee, Jinho Lee, In-Hyuk Ha.

**Writing – original draft and revision:** Kyoung Sun Park.

## Supplementary Material

Supplemental Digital Content
