# Supplementary figures and images for: A study on the effectiveness of pharmacopuncture for chronic neck pain: A protocol for a pragmatic randomized controlled trial
Source: Medicine (Baltimore). 2020 Jul 31;99(31):e21406. doi: 10.1097/MD.0000000000021406 (PMC7402875; doi:10.1097/MD.0000000000021406)

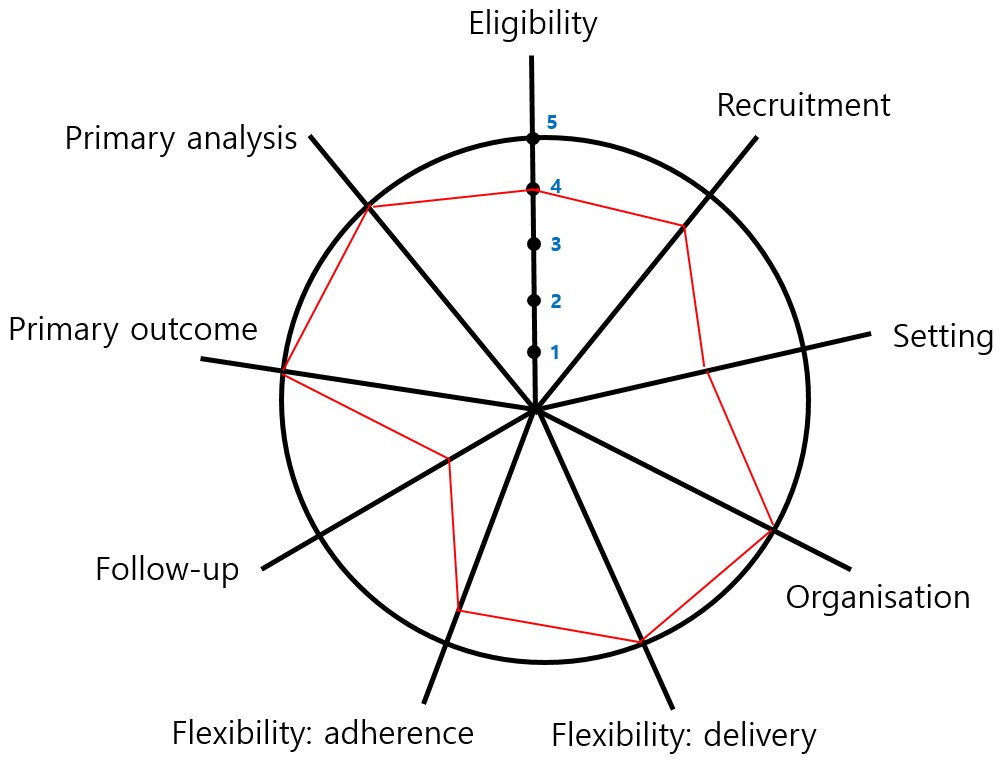

Supplement: Supplemental Digital Content [file medi-99-e21406-s001.jpg]
